# Treatment optimization by monitoring vancomycin concentration in the serum and cerebrospinal fluid in a child with cystoperitoneal shunt-related infection caused by methicillin-resistant *Staphylococcus aureus*: a case report and literature review

**DOI:** 10.1007/s00381-023-06004-0

**Published:** 2023-06-05

**Authors:** Shinsuke Mizuno, Junji Koyama, Hiroshi Kurosawa, Masashi Kasai

**Affiliations:** 1 Department of Pediatrics Infectious Disease, Hyogo Prefectural Kobe Children’s Hospital, 6500047 Kobe City, Hyogo, Japan; 2 Department of Neurosurgery, Hyogo Prefectural Kobe Children’s Hospital, Kobe City, Hyogo, Japan; 3 Department of Pediatric Critical Care Medicine, Hyogo Prefectural Kobe Children’s Hospital, Kobe City, Hyogo, Japan

**Keywords:** Therapeutic drug monitoring, Methicillin-resistant *Staphylococcus aureus*, Cysto-peritoneal shunt

## Abstract

**Background:**

Cerebral ventricular shunt infections caused by methicillin-resistant Staphylococcus aureus (MRSA), especially strains with elevated minimum inhibitory concentration (MIC) values, have a poor prognosis. Monitoring serum vancomycin (VCM) levels with therapeutic drug monitoring and maintaining high VCM concentrations in the cerebrospinal fluid (CSF) are critical to treatment success. However, there have been a few reports about the CSF penetration and the pharmacokinetics of VCM in children.

**Case presentation:**

Here, we report the case of a pediatric patient with cysto-peritoneal shunt-related meningitis caused by MRSA with an MIC of 2 μg/mL. The adequate VCM concentration was maintained by monitoring the VCM concentration in the CSF via the external ventricular drain, and frequent blood taking was avoided. VCM showed a good CSF penetration in our patient, and she was discharged without complications.

**Discussion:**

Therapeutic drug monitoring of VCM concentration in the CSF may result in successful treatment even if MRSA shows a higher MIC. Therapeutic drug monitoring of VCM concentration in the CSF may also reduce the side effects.

## Background

Post-neurosurgical central nervous system (CNS) infections can occur in 1%–8.6% of cases and can be treated, but antimicrobial-resistant pathogens are a growing concern and limit treatment options [1–4]. Therapeutic drug monitoring (TDM) is recommended to ensure appropriate antimicrobial therapy for antimicrobial agents with a narrow range of efficacy and toxicity. The drug concentration is measured, and the optimal dosage and administration method are established based on pharmacokinetics/pharmacodynamics theory [5].

Vancomycin (VCM) has a time-dependent activity with limited penetration in the cerebrospinal fluid (CSF) because of its hydrophilicity and large molecular size [5]. Therefore, a high serum concentration is needed to achieve an appropriate CSF concentration. However, the penetration of VCM is variable and unpredictable, depending on patient factors [6, 7].

The recent guidelines recommend VCM for treating CNS infections caused by methicillin-resistant *Staphylococcus aureus* (MRSA). However, these recommendations are primarily based on data from adult populations. They also propose a second-line drug if the strain’s minimum inhibitory concentration (MIC) value is 2 μg/mL [1].

Here, we report the case of a pediatric patient with cysto-peritoneal shunt-related infection caused by MRSA with MIC value of 2 μg/mL. We successfully treated with VCM by monitoring both CSF and serum concentration levels of VCM.

## Case presentation

A 2-year-old girl with congenital intracranial cysts underwent endoscope-assisted fenestration and shunt valve replacement surgery against cysto-peritoneal shunt dysfunction. The pregnancy was uneventful and the child was born normally at 3,214 g, 49.0 cm height, and 43.1 cm head circumference. She had congenital intracranial cysts and a cerebral malformation was detected by pathological examination at 8 days of age. A cysto-peritoneal shunt was inserted 1 month after birth.

Four days after the operation (day 1), she developed a fever and irritability. On physical examination, she was febrile, had a Glasgow Coma Scale of E3V5M6, and showed nuchal rigidity. The patient’s laboratory data were white blood cell count, 13.7 × 10^9^/L and C-reactive protein, 19.4 mg/dL. CSF examination showed a cell count of 236 /μL; total protein, 10.6 g/L; and glucose, 2.6 mmol/L. Gram staining of the CSF detected gram-positive cluster microorganisms. We initiated intravenous VCM. MRSA was isolated from the CSF on day 2; the MIC of VCM was 2 μg/mL (Table 1). The cysto-peritoneal shunt was removed, and an external ventricular drain (EVD) was placed on the same day. A shunt culture was also positive for MRSA.

**Table 1:** Antimicrobial susceptibility profile of *Staphylococcus aureus* isolated from the blood and cerebrospinal fluid of the patient

**Antimicrobial**	**MIC (mcg/mL)**	**Interpretation**
Penicillin	> 8	R
Ampicillin	> 8	R
Cefazolin	16	R
Imipenem	4	R
Gentamicin	< = 2	R
Erythromycin	> 4	R
Levofloxacin	> 4	R
Sulfamethoxazole/Trimethoprim	< = 1	R
Vancomycin	2	S
Teicoplanin	< = 2	S
Linezolid	2	S
Daptomycin	0.5	S
Clindamycin	> 2	R
Minocycline	> 8	R
Rifampicin	< = 0.5	S

*S* susceptible, *R* resistant

The VCM levels in the serum and CSF were measured during treatment (Table 2). The dosage of VCM was adjusted to achieve both a CSF trough level of at least 2.0 mg/L and a serum trough level of less than 20 mg/L, resulting between 70 − 100 mg/kg/d. The median serum trough level was 13.7 mg/L (interquartile range [IQR]: 10.7 − 15.2 mg/L), and the median CSF trough level was 5.3 mg/L (IQR: 4.1 − 5.9 mg/L), and the median CSF/serum concentration ratio was 0.29 mg/L (IQR: 0.28 − 0.42 mg/L). Her serum creatinine levels showed a normal range during treatment.

**Table 2:** Vancomycin dose and concentration in the serum and cerebrospinal fluid collected from the extraventricular drain during treatment

	Day 4	Day 5	Day 6	Day 8	Day 9	Day 10	Day 12	Day 14	Day 17	Day 20
Serum (mg/L)	7.9	8.4	10.7	20.1	13.7	11.3	15.2	14.6		16.4
CSF (mg/L)			4.6	5.9	3.9			3.9	5.9	6.9
Serum / CSF			0.43	0.29	0.28			0.27		0.42
Dose (mg/kg/d)	80	90	100	75	75	75	72	72	72	70
Cre (mg/dL)	0.19		0.20	0.23	0.28	0.25	0.26	0.26		0.28

*CSF* cerebrospinal fluid, *Cre* creatinine

On day 3, her symptoms improved. CSF culture was no MRSA growth shown on day 6. On day16, brain magnetic resonance imaging was performed, and no findings of abscess formation showed. Antibiotic treatment was ended on day 29, and the patient was discharged without any complications on day 42.

## Discussion

We successfully treated cysto-peritoneal shunt-related infection caused by MRSA with MIC of VCM of 2 μg/mL by monitoring both CSF and serum concentration levels of VCM. The penetration rate of VCM from the blood to CSF was shown to be sufficiently high.

The target concentration for CNS infection caused by MRSA with MIC of VCM of 2 μg/mL could not be achieved based on the in vitro data [6]. However, the guideline states that VCM can be continued if the patients improve clinically because one point of MIC difference can occur by laboratory error and the MIC result varies based on the method used [1, 8–11].

There has been no clear evidence of the safety and efficacy levels of VCM concentration in CSF. However, based on the data from intraventricular administration, the CSF trough levels > 10 times the MIC have not been associated with severe or irreversible adverse events [1]. Concerning indicators of efficacy, the CSF trough concentration above the MIC has been suggested in pediatric patients [8].

The penetration rate has been reported in the range of 0 − 68% in children (Table 3) [12–15]. A higher penetration rate by opening of the tight junctions of the blood–brain-barrier cells, delayed drug removal by a decrease of the CSF bulk flow, and inhibited activity by efflux pump of antibiotics have been occurred during the acute phase of bacterial meningitis [6, 7, 15, 16]. Otherwise, intense inflammation was not regularly present in cerebral ventricular shunt-related infections [7, 17]. However, there have been some reports that the patients with cerebral ventricular shunt- or EVD-related infection showed relatively higher levels of antibiotics concentration in the CSF than those without these devices because of the disruption of the blood-CSF barrier [14, 18–20].

**Table 3:** Pharmacokinetics of vancomycin in pediatric patients with central nervous system infection

Study number	Study place	Number of patients	Age	Dosage of VCM	Concentration in CSF	CSF/serum (%)	Reference, first author (year)
1	USA	6	11 years (mean)	10–20 mg/kg, every 6-12 h	2.48 (mean)	0.77–18 (rang)	Laney Jorgenson (2007)
2	South Africa	9	15 months (mean)	15 mg/kg, every 6 h	3.3 (mean)	21 (mean)	K P Klugman (1995)
3	USA	3	26–31 weeks of gestation (range)	20 mg/kg, every 18-24 h	2.2–5.6 (range)	26–68 (rang)	Review P D Reiter (1996)
4	USA	8	4.3 years (median)	19 mg/kg, every 8 h (median)	1.07 (median)	8 (median)	Julie Autmizguine (2014)
5	Japan	1	2 years	70–100 mg/kg/d (range)	5.3 (median)	29 (median)	

*CSF* cerebrospinal fluid, *VCM* vancomycin

Several studies have reported that intraventricular use of VCM may improve treatment outcomes without severe side effects in adult patients [21–23]. However, arecent systematic review noted insufficient evidence in pediatric patients, and intraventricular antimicrobial therapy is considered when clinical improvement is poor with intravenous administration alone [24].

In our patient, although the strain isolated from the CSF showed MIC of VCM of 2 μg/mL, successful treatment with intravenous VCM was achieved by monitoring the concentration in both the serum and CSF. The penetration rate was sufficiently high and the CSF trough levels were above the MIC during treatment.

High serum VCM concentration can cause complications such as nephrotoxicity, ototoxicity, and vasculitis [4]. We could avoid unnecessary dose increases by monitoring the CSF concentration, which may lead to excellent tolerance and no clinically significant adverse events.

In conclusion, monitoring the VCM concentration in the CSF and its serum concentration as indicators may help make decisions about the optimal dosage, changing second-line drugs, and reducing the frequency of side effects.

## Data Availability

The datasets generated and/or analyzed during this study are available from the corresponding author upon reasonable request.

## List of abbreviations

CNS: Central nervous system
CSF: Cerebrospinal fluid
EVD: External ventricular drain
IQR: Interquartile range
MRSA: Methicillin-resistant *Staphylococcus aureus*
MIC: Minimal inhibitory concentration
TDM: Therapeutic drug monitoring
VCM: Vancomycin
